# Adult-onset Still’s disease with concurrent acute necrotizing encephalopathy: a case report

**DOI:** 10.1186/s12883-022-02844-6

**Published:** 2022-09-01

**Authors:** Xue Yang, Meiling Wei, Shuguang Chu, Yue Zhang

**Affiliations:** 1 Department of Rheumatology, Huashan Hospital, Fudan University, Shanghai, China; 2 Department of Neurology, Donglei Brain Hospital, Shanghai, China; 3 Department of Radiology, Shanghai East Hospital, Tongji University School of Medicine, Shanghai, China; 4 Department of Neurology, Shanghai Huashan Hospital, Fudan University, Jing’an District, No. 12 Mid Urumqi Road, Shanghai, 200040 China

**Keywords:** Acute necrotizing encephalitis, Adult-onset Still’s disease, Hemorrhage, Comorbidity

## Abstract

**Background:**

Acute necrotizing encephalopathy (ANE) is a rare encephalopathy characterized by multiple symmetrical brain lesions, mainly involving thalami. Adult-onset Still’s disease (AOSD) is a rare systemic inflammatory condition of unknown cause characterized by fever, sore throat, rash and joint pain. Both entities are considered to be triggered by infections and associated with hypercytokinemia.

**Case presentation:**

A 46-year-old male was diagnosed with AOSD at local hospital because of 3-week-long high fever, sore throat, arthralgia, transient skin rash, lymphadenopathy, leukocytosis, hyperferritinemia, and absence of antinuclear antibodies (ANA) and rheumatoid factor (RF). Corticosteroids were not used because of delayed diagnosis. Three weeks after the onset, the patient suddenly fell unconscious and was transferred to our hospital. Brain CT and MRI revealed symmetrical lesions involving thalami, striatum and brain stem, consistent with ANE. One day after admission, his condition aggravated and brain CT revealed hemorrhage in the lesions. He died 3 days after admission.

**Conclusion:**

We report a rare case of ANE preceded by AOSD. The underlying mechanism is still unclear. Early recognizing of the two conditions is difficult but prognostically important.

## Background

Acute necrotizing encephalopathy (ANE) is a rare type of encephalopathy characterized by symmetrical brain lesions involving thalami, putamina, cerebral and cerebellar white matter, and brain stem tegmentum [1]. Though the pathogenesis of ANE is unclear, it is generally considered to be a parainfectious encepalopathy [1] and “cytokine storm” may play a role in the development of ANE [2]. Adult-onset Still’s disease (AOSD) is a rare systemic inflammatory condition of unknown etiology characterized by high fever, transient skin rash, arthralgia/arthritis, sore throat, lymphadenopathy, hepatomegaly or splenomegaly, leukocytosis, abnormal liver function tests, hyperferritinemia and absence of antinuclear antibodies (ANA) and rheumatoid factor (RF) [3]. AOSD is also associated with infections and hypercytokinemia [4]. Here, we report a case of ANE accompanied with AOSD. To the best of knowledge, this is the first case of AOSD complicated with ANE in the literature.

## Case presentation

A 46-year-old male developed fever of 37.8℃, sore throat, and pain and swelling in elbows, wrists, knees and ankles 3 weeks before admission. Physical examination found grade 1 tonsils and laboratory tests revealed leukocytosis of 15.79 × 10^9/L (85.5% neutrophils) at the onset. He was treated for acute pharyngitis with cephalosporin and penicillin for 11 days but symptoms did not resolve. He was admitted to the local hospital 10 days ago because of fever of up to 40.1℃, accompanied with skin rash which was more conspicuous during febrile episodes. Blood tests showed elevated inflammatory markers but did not find clues of infections or autoimmune diseases (Table 1). Tumor markers, vitamins levels and thyroid function were normal. Bone marrow aspiration showed no findings of phagocytosis. CT scans of the thorax and abdomen were unremarkable. During admission, he was treated on an empirical basis with piperacillin/tazobactam, moxifloxacin, imipenem/cilastatin, teicoplanin, tigecycline and meropenem sequentially but were all of no avail. AOSD was thus suspected. However, he suddenly fell unconscious 14 h ago and was transferred to our hospital. Glucosteroids was not used as planned because of delayed diagnosis of AOSD.

**Table 1:** The evolution of the laboratory test results carried out at the local hospital (on 03 September 2021) versus our hospital (on 13 September 2021)

	03 Sept 2021	13 Sept 2021	Reference range
Leukocytes	12.45	4.11	3.5–9.5*10^9/L
Hemoglobin	122	127	130-175 g/L
Platelets	277	171	125–350*10^9/L
CRP	147	151.48	< 10 mg/L
ESR	75	ND	< 20 mm/h
ALT	28	75	9-50U/L
AST	30	124	15-40U/L
Albumin	24.7	26.9	40-55 g/L
Na +	128	128.1	137-147 mmol/L
LDH	649	2680	109-245U/L
Ferritin	≥ 2000	≥ 2000	15-200 ng/mL
PT	15	13.5	11-13 s
Fibrinogen	6.86	4.17	2-4 g/L
D-dimers	1070	10,000	< 200ug/L
Lactate	1.19	1.29	0.5–1.7 mmol/L
Ammonia	ND	45	18-72umol/L
Tests for infections			
HIV	negative	negative	negative
EBV	negative	ND	negative
Syphilis	negative	negative	negative
HBV	positive (462.57 ng/ml)	ND	negative
Blood culture	negative	ND	negative
G-test	negative	ND	negative
GM-test	negative	ND	negative
T-SPOT	negative	ND	negative
NGS	negative	negative	negative
Autoimmune antibodies			
RF	negative	negative	negative
ANA	negative	negative	negative
Anti-dsDNA	negative	negative	negative
ASO	negative	negative	negative
Anti-GBM	negative	negative	negative
ANCA	negative	negative	negative
ACA	negative	negative	negative
Anti-CCP	negative	negative	negative

*CRP* C-reactive protein, *ESR* Erythrocyte sedimentation rate, *ND* Not done, *LDH* Lactate dehydrogenase, *PT* Prothrombin time, *HIV* Human immunodeficiency virus, *EBV* Epstein–Barr virus, *HBV* Hepatitis B virus, *GM* Galactomannan, *NGS* Next generation sequencing, *ASO* Anti-streptolysin O antibody, *GBM* Glomerular basement membrane antibodies, *ANCA* Antineutrophil cytoplasmic antibodies, *ACA* Anticardiolipin antibodies, *CCP* Cyclic citrullinated peptide antibody

**Fig. 1:**
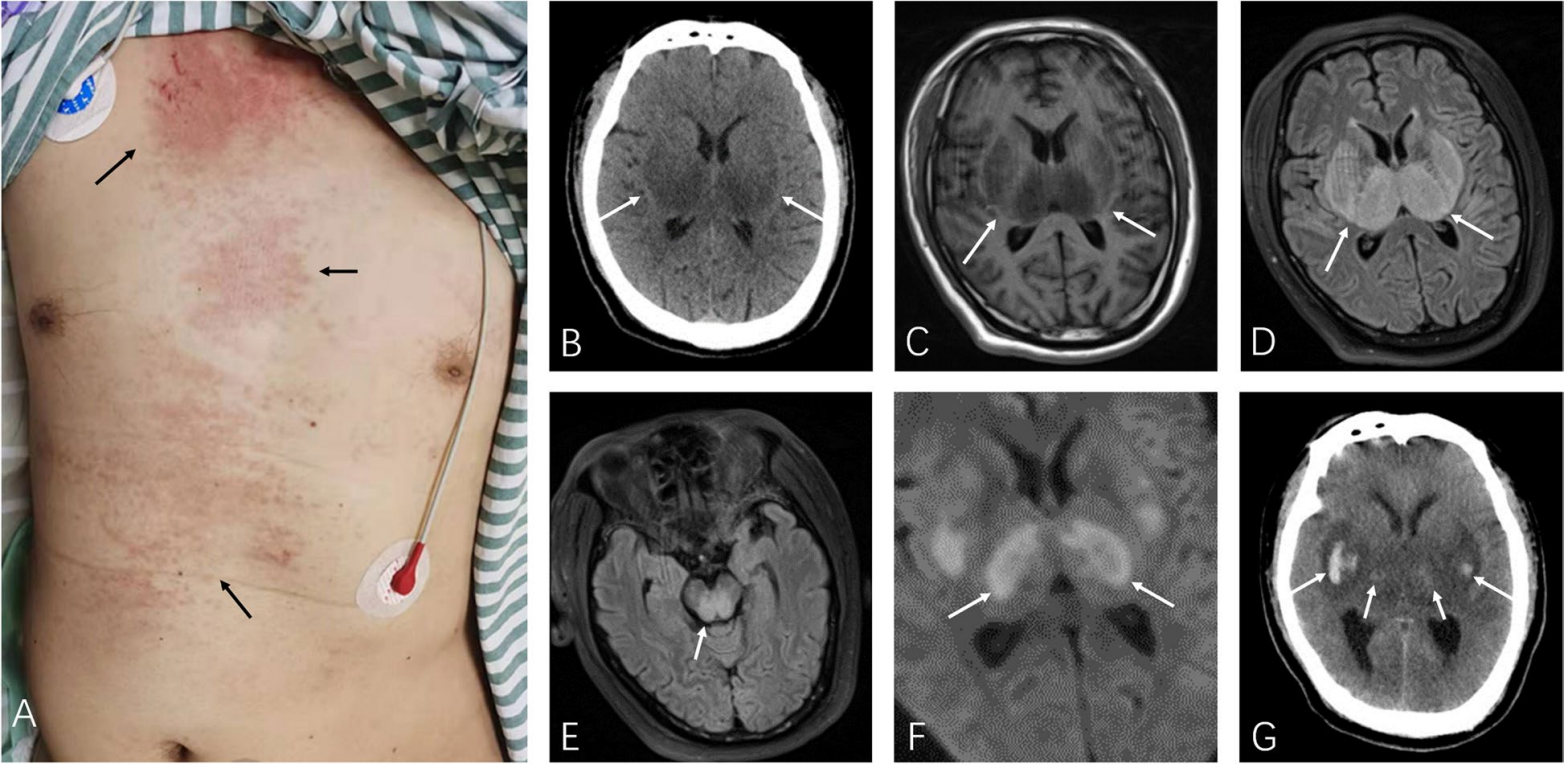
Picture of the patient (**A**) showing salmon-colored rash in the trunk (black arrows). On hospital day (HD) 1, brain CT (**B**), T1 weighted (**C**), and T2-FLAIR (**D**, **E**) MRI demonstrating symmetric lesions involving thalami, striatum and brain stem, consistent with acute necrotizing encephalopathy (white arrows). Diffusion weighted images (**F**) showing typical “concentric/laminar structure”. Brain CT on HD 2 revealing hemorrhage in the lesions (**G**, white arrows)

On admission, vital signs were as follows: T: 38℃, HR: 87 bpm, R: 18 bpm, BP:117/86 mmHg, SPO_2_: 98%. Physical examination found patchy salmon-colored skin rash in the trunk and thighs (Fig. 1A). Enlarged axillary lymphadenopathy was detected but with no hepatosplenomegaly. Glasgow coma scale was graded 4 (E1V1M2). Pupils were symmetric in size but unreactive to light. Neck was stiff. Babinski signs were positive bilaterally. Laboratory tests results were listed in Table 1. Lumbar puncture revealed opening pressure of 130mmH_2_O. Cerebrospinal fluid (CSF) analysis showed elevated erythrocyte count to 120 × 10^6/L with normal leukocyte count. CSF glucose, protein and chloride levels were normal. Cultures, cytology and next generation sequencing (NGS) of CSF were negative. Brain CT and MRI revealed lesions involving bilateral striatum, thalamus, and brain stem, typical for ANE (Fig. 1B-F). Brain digital subtracted angiography (DSA) was conducted to rule out venous thrombosis and other cerebral vascular diseases. His condition deteriorated rapidly and was intubated on hospital day 2. A repeat brain CT revealed hemorrhage in the lesions (Fig. 1G). ANE accompanied with AOSD was considered. However, his relatives gave up treatment and the patient passed away on hospital day 3.

## Discussion and conclusion

ANE is a rare form of encephalopathy which was first reported by Mizuguchi et al*.* in 1995 [1]. It is more commonly seen in children than in adults without racial predilection. Most cases of ANE are sporadic but familial ANE which is caused by the missense mutations in the *RANBP2* gene has been reported [5]. Infection is a common trigger for ANE. Viral prodrome usually precedes neurological deficits, but pathologically, the lesions show edema, hemorrhage, necrosis with few inflammatory cells, suggesting blood–brain barrier breakdown rather than direct viral invasion or parainfectious demyelination. Because clinical manifestations lack specificity, the diagnosis of ANE is mainly based on characteristic neuroradiologic findings, that is, multifocal and symmetric brain lesions, mainly involving bilateral thalami. The pathogenesis of ANE has not been fully elucidated. The most prevalent hypothesis is “cytokine storm”, involving interleukin- (IL-) 6, tumor necrosis factor-alpha (TNF-α), IL-10, IL-15, IL-1β, and soluble TNF receptor [2]. IL-6 was neurotoxic at high concentrations and TNF-α could damage the endothelium of the central nervous system [2]. The differentials include Leigh syndrome, thrombosis of the internal cerebral veins or straight sinus, Wernicke encephalopathy and Japanese encephalitis. In the present case, vitamins, glucose, lactate and ammonia levels were all normal. Cerebral veins thrombosis was ruled out by DSA. Infections were excluded by many laboratory methods and unsuccessful empirical antibiotics treatment. Treatment of ANE is mainly supportive. Intravenous glucocorticoids, immunoglobulin, and plasmapheresis were tried in previous cases but prognosis is still very poor [2]. Steroid within 24 h after the onset was related to better outcome in children with ANE without brainstem lesions [6], so steroid treatment should be initiated as early as possible.

AOSD is an uncommon systemic auto-inflammatory disease. In our case, the presentations and laboratory findings supported the diagnosis of AOSD according to criteria proposed by Yamaguchi et al. [3]. Similar to ANE, AOSD may also be triggered by viral or bacterial infection and pathogenesis also involves “cytokine storm”. Relevant proinflammatory cytokines such as IL-1β, IL-6, IL-8, TNF-α, and IL-18 [7] largely overlap with those found in ANE [2]. Despite the theorical possibility of concurrence of ANE and AOSD, similar literature is few. Zhao M, et al.reported neurological complications occurred in 7.5% patients with AOSD and aseptic meningitis was the most common presentation [8]. It is believed that in the hyperinflammatory state, activated neutrophils in peripheral blood can cross the blood–brain barrier and induce meningitis [8]. Cerebral infarction [8], cerebral hemorrhage [9], encephalitis [8], demyelinating encephalopathy [10], and reversible posterior leukoencephalopathy syndrome [11] were anecdotally reported. Up to our knowledge, this is the first case report of coexistence of AOSD and ANE. We assume the rarity in clinical practice may be due to the diagnostic challenges in both conditions. The diagnosis of AOSD is highly dependent on the physician's judgment and is generally an exclusive one. The median interval between onset of symptoms and a definite diagnosis of AOSD ranged between 1 and 4.1 months [12]. As for ANE, it typically develops in children younger than 5 years of age; very few case reports have been published in adults [13], so it is an under-recognized syndrome. Delayed diagnosis of AOSD in our case hampered timely immunotherapy and cytokine storm became so severe that ANE followed.

There were two limitations in this case report. AOSD is an exclusive diagnosis that needs to exclude infections, tumors, and rheumatic diseases. In the present case, laboratory studies and failure of empirical biotics treatment did not support infections or other rheumatic diseases, but underlying tumors were difficult to be ruled out. Lymphoma [14], leukemia [15], breast cancer [16], ovarian cancer [17], and thyroid cancer [18] can give rise to AOSD-like symptoms. Although bone marrow aspiration and CT scans of thorax and abdomen were performed in this case, a total-body PET-CT scan was indicated. The other pitfall is macrophage activation syndrome (MAS) could not be completely excluded in this case. MAS is a severe inflammatory systemic abnormality with lethal potential characterized by pancytopenia, coagulopathy, hepatopathy, neurological disorders and hemophagocytosis [19]. It develops in 10.2% patients with AOSD and has an association with an increasing risk of neurological manifestations [8]. ANE has been reported in cases with hemophagocytic lymphohistiocytosis (HLH) [20, 21]. Although our patient had no fulfilment of criteria for the diagnosis of HLH [22] due to the absense of splenomegaly, cytopenia, hypertriglyceridemia, and hemophagocytosis initially, HLH was still possible in the late stage and a repeat bone marrow aspiration was necessary. However, the illness progressed so rapidly that scheduled PET-CT and bone marrow aspiration were not able to be performed.

In conclusion, we report a case of ANE associated with AOSD. Physicians should be aware of this uncommon but life-threatening complication when patients with AOSD developed conscious disturbance. More observations are warranted and the underlying mechanism needs to be elucidated.

## Data Availability

Not applicable.

## Abbreviations

ANE: Acute necrotizing encephalitis
AOSD: Adult-onset Still’s disease
ANA: Antinuclear antibodies
RF: Rheumatoid factor
CSF: Cerebrospinal fluid
NGS: Next generation sequencing
DSA: Digital subtracted angiography
IL: Interleukin
TNF: Tumor necrosis factor
MAS: Macrophage activation syndrome
HLH: Hemophagocytic lymphohistiocytosis
